# Bayesian predictive system for assessing the damage intensity of residential masonry buildings under the impact of continuous ground deformation

**DOI:** 10.1038/s41598-024-82038-x

**Published:** 2025-01-09

**Authors:** Janusz Rusek, Leszek Chomacki, Leszek Słowik

**Affiliations:** 1 Faculty of Geo-Data Science, Geodesy, and Environmental Engineering, AGH UST of Krakow, 30059 Cracow, Poland; 2 Building Research Institute, 00611 Warsaw, Poland

**Keywords:** Building damage, Damage prediction, Bayesian network, Mining, Continuous deformations, Civil engineering, Computational science, Natural hazards

## Abstract

The paper introduces a method for predicting damage intensity in masonry residential buildings situated in mining areas, focusing on the impact of large-scale continuous ground deformation. The research utilizes in situ data collected in a database, encompassing structural and material features, as well as information on maintenance quality and building durability. In addition to this information, the database collected data on the intensity of continuous deformation of the mining area at the location of the building, as well as the range and intensity of damage identified in buildings. The information included in the database was the result of many years of observations of buildings during the disclosure of impacts from mining exploitation and was based on: the results of in-situ building inventory, analysis of available building documentation and information provided by mining companies. The archived data were categorized variables labeled. The transformation of the data to a labeled value was dictated directly by the assumptions of the GOBNILP algorithm. Ultimately, a predictive model, represented by an optimal Bayesian network structure, is established. The optimisation of the network structure is achieved through the adaptation of the GOBNILP Bayesian network learning algorithm from data. This optimisation process is executed through the Gurobi Optimizer. It is worth noting that this interdisciplinary approach represents one of the first applications of such a methodology in the field of civil and environmental engineering. The results obtained can therefore be of significant value given the fact that the methodology of detecting the structure of Bayesian networks from data is still developing intensively in other scientific fields. In the course of the analyses, metric scores are examined, and various network structures are assessed based on their complexity. Great values of classification accuracies over 91% were obtained. This meticulous evaluation allows for the selection of the optimal Bayesian network that best generalises the knowledge acquired during the learning process. The paper also demonstrates the potential application of the obtained model in diagnosing damage causes and predicting future occurrences, highlighting the versatility of the proposed approach for addressing issues in the field.

## Introduction

Damage to buildings not only poses a threat to the safety of the structure and their occupants, but also contributes to a reduction in their utility value, including the deterioration of thermal performance^1^. The process of damage is an inherent feature that occurs during the life cycle of buildings. There are many reasons for this process. These are primarily design and workmanship errors, improper use, including lack of ongoing maintenance and repair, and exposition to negative external environmental impacts. Here, the influences can be divided into chemical, biological, and mechanical groups. The problem addressed in this paper focuses on mechanical impacts induced by mining and post-mining activities. In mechanistic terms, these represent kinematic excitations of the foundations of buildings, determining additional force on their structural elements.

At present, many buildings, including residential buildings of masonry or reinforced concrete construction, are located in industrialised sites that are mining areas of underground mines^2–4^. These buildings, which are permanently subjected to mining impacts, are affected by accelerated degradation due to, among other things, the combined effects of material ageing and the formation of damage in structural and secondary components. These impacts usually take the form of large-scale subsidence^5–7^ and mining tremors^8–10^. In order to prevent this degradation, assessments of the degree of hazard to buildings are carried out in terms of the potential for their structure to absorb negative impacts from predicted or ongoing mining exploitation^11–14^. Unfortunately, these methods are ineffective, too general and therefore represent reality in a very approximate manner.

The actual damage process is determined by a number of factors that need to be captured when assessing the threat to buildings^15,16^. The need to carry out such an assessment for a very large number of buildings located in a mining area makes it inefficient, if not impracticable, to apply a numerical FEA approach to each of them. In addition, the issue is complicated by the need to take into account factors such as the age of the building, how it is maintained, etc. These are non-technical parameters yet are necessary to consider the behaviour of the structure throughout its technical life cycle.

The need to take into account typically technical parameters such as structural and material features and the construction solutions used together with non-technical parameters makes the problem multidimensional. With data from many years of observation, an attempt can be made to establish a model of such a complex process in empirical form. Here, it seems indispensable to apply methods that allow the creation of multidimensional models while taking into account nonlinearity and uncertainty. Taking this into account, the team of authors has made attempts to do so in the past years using selected machine learning methods^2,17–19^. In these studies, predictive models for the damage process were obtained, which were characterised by good fitting properties to the learning data and an acceptable level of generalisation of the acquired knowledge. It should be noted that the obtained models constituted a predictive tool, acting in one direction and indicating the predicted intensity of damage in buildings for given conditions resulting from mining impacts. As a result of the method used, structural reinforcements can be undertaken in advance, the implementation of which should minimise the risk of a structural failure situation. In addition to the static-strength aspect, the economic factor is also important. This is because, very often, an in situ damage situation generates a major socio-economic problem. This mainly involves claims for compensation from mining companies for damage to buildings. With this in mind, a review of methods has been carried out to satisfy the above-mentioned requirements, but also to enable the model to work in the reverse situation of the prediction, i.e. the diagnosis of causes for damage that has occurred. The best tool for solving such a task turned out to be the use of Bayesian network methodology^20^.

Bayesian networks allow a multi-parameter representation of the damage process giving the possibility of multi-directionality^4,21^. In a large generalisation, this involves the fact that, given a Bayesian network structure, it is possible to infer the state that any node in the network takes^22–25^. This feature allows the construction of a model that can perform both a prognostic and diagnostic role, as required.

Bayesian networks have been successfully applied to problems related to predicting the consequences of natural disasters^26–31^. This area is most similar to the problem addressed in the paper, although in the case of buildings in mining areas, damage rarely leads to catastrophic consequences. Here it is a more subtle issue but one that carries broad socioeconomic repercussions, making it an overly complex problem. The use of Bayesian networks in the context of damage assessment or failure prediction is also quite common for underground linear structures such as pipelines^32–37^ or tunnels^38,39^. These are also related issues to the one dealt with in this work due to a similar mechanism of transferring impacts from the ground, taking place through friction, analogous to the transfer of continuous ground deformations to the foundations of buildings. Similarly, in the context of strictly geotechnical issues, one can also find research results justifying the use of Bayesian networks^40–44^. This type of methodology is also used extensively in issues of assessing the reliability of building structures^45–50^.

In addition to the above-mentioned area related to estimating the risk of natural disaster impacts, Bayesian networks have been used for many years in medicine^51–54^, genetics^55,56^, biology or economic^57–59^ and social^60^ areas. It is on the ground of data from these areas that algorithms dedicated to parameter learning and structure extraction of the Bayesian network from data are often validated.

As can be seen, the implementation of the Bayesian networks methodology can be observed in a number of cases that to some degree relate to the research subject undertaken within the framework of this work. Therefore, this justifies the implementation of this methodology also for the damage prediction of masonry buildings subjected to the impacts of continuous ground deformation. Nevertheless, the referred cases mostly describe a situation in which the structure of the Bayesian network is arbitrarily given by the user, and only the parameters of the so-called conditional probability tables are subjected to tuning, if any. On this background, the methodology used in the paper, dealing with learning the structure of the Bayesian network on the basis of data, appears as an extension of previously used solutions in the interdisciplinary field of civil engineering, mining and environment. Thus, it can be assumed that the results presented in the paper make a novel contribution to these research areas.

The main difficulty with creating a Bayesian network is correctly predicting its structure^2,22^. For low-complexity issues, the structure can be predicted and given arbitrarily. However, attempting to map multivariate issues requires first the determination of the optimal structure of the Bayesian network and then tuning of its parameters^61^. In this issue, the most challenging part is to extract the network structure. This is done on the basis of the data. For this purpose, dedicated Bayesian Network Structure Learning (BNSL)^61^ algorithms are used, which, acting on the principle of optimisation, allow the optimal Bayesian network structure to be extracted. Unfortunately, most BNSL algorithms use local optimisation methods, which do not guarantee the optimal Bayesian network structure in a global sense.

In recent years, a method has been developed to discover the optimal structure of Bayesian networks globally. This method based on Integer Linear Programming (ILP)^62^ optimisation called Globally Optimal Bayesian Network learning using Integer Linear Programming (GOBNILP)^63,64^ provides a perspective on how to solve a pre-posed question in global terms. This particular method was selected for the study, the results of which are described in this article.

It should be noted that Bayesian networks are also considered to be generative Artificial intelligence (AI) tools^65–67^. By the fact that the structure of the network represents a joint probability distribution, they allow the generating of new data, similar to those used in the learning process. This offers the possibility of extending the research horizon, based on the concept of data augmentation (DA^68,69^), in order to increase the amount of data, which will consequently make the empirical knowledge of the damage process more detailed and precise.

The issue under consideration has a very strong utilitarian aspect. The research presented will be discussed using the example of real data obtained from a long-term inspection of buildings with traditional masonry construction. This group is the most common type of structure in highly urbanised areas influenced by mining exploitation. Damage to masonry buildings is a very widespread problem affecting many property owners and supervisors. It is also relevant to the maintenance management of buildings and the optimal planning of rehabilitation works. The Bayesian network tool itself can be understood as a multivariate decision support system that can be successfully implemented within Structure Health Monitoring (SHM)^70^.

## Description of database and specification of mining impacts

This chapter presents the structure of the building database. Basic information is provided on their structural and material solutions and the intensity of damage found during the in situ inspection. In the following section, the types of impact resulting from the formation of the mining basin are briefly characterised. Reference is also made to other possible symptoms of mining exploitation manifested on the surface of the mining area.

### Characteristics of collected database

**Fig. 1:**
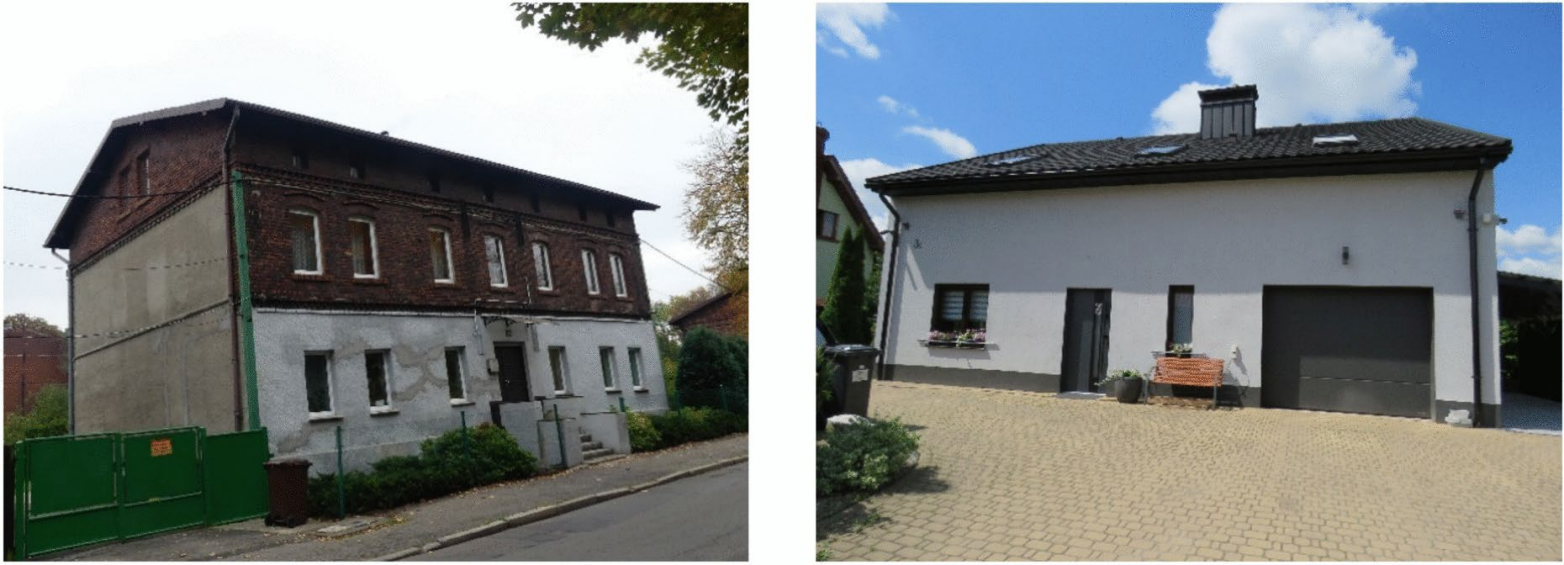
Examples of residential masonry building types (examples from the database).

For the basic analysis, a qualified group of 207 buildings was selected. Information about these buildings was gathered during field research and organised in a database. The exemplary buildings from this database, which serve as the foundation for this article, are illustrated in Fig. 1.

The multi-family residential buildings were built between 1900 and 1940. They were built as free-standing or compact buildings. They usually have a basement. These buildings have two to four above-ground floors.

Their foundations and basement walls are made of stone or brick, and the above-ground walls are made of brick. Ceilings above basements are usually made of brick on steel beams, sometimes as vaults. Ground floors usually have wooden ceilings. Lintels are brick vaulted or flat. Stair structures within the staircases are made of wood and steel. The analysed buildings are protected against mining impacts in the form of full or partial anchoring with steel rods in the ceiling levels.

Single-family buildings, characterised by their low-rise nature with a height of up to two above-ground storeys, were constructed using traditional brick technology before 2017. These structures are typically free-standing or semi-compact in design.

Single-family houses are characterised by greater construction diversity. The height of the buildings is up to two floors above ground. Older structures were constructed similarly to the multi-family buildings described above.

The buildings erected after 1950 were made on reinforced concrete foundations. The basement walls are made of brick or concrete blocks. The walls on the ground floor are made of brick, aerated concrete blocks or ceramic blocks. The ceilings above the basement are made of concrete on steel beams or reinforced concrete. The ceilings of the upper floors are made of rib-and-slab concrete or reinforced concrete. Lintels are made of steel beams or reinforced concrete. Structures were designed to absorb mining influences, characteristic of category III or IV mining areas, in the form of reinforced concrete benches and reinforced concrete ceilings with peripheral rings.

After repeated inspections of the technical condition of 207 buildings, research material was collected covering 594 cases. A summary of geometric parameters, construction parameters, and mining impacts is presented in Table 1.

**Table 1:** List of variables with indication of their discretisation (categories).

Variable type	Variable	Code	No. of categories
Geometry	Building length	Length	8
	Building width	Width	6
	Building area	Area	10
	Number of above-ground storeys	NoSto	5
	Building volume	Volume	11
	Length of a row of compact buildings	LenRow	12
	Method of dilation	Dilat	3
	The shape of the building	Shape	4
	Type of basement	Base	3
	Variable level of foundation	VarFou	2
	Variable building height	VarHei	2
Construction	Type of foundation	Found	3
	Basement wall material	BWM	3
	Material of the walls of the ground floor and above	GWM	2
	Ceiling above the basement	CeiBas	5
	Ceiling above the ground floor and above	CeiGro	3
	Type of lintels	Lintel	3
	Structural protections for mining influence	Secur	4
	Structural protections—supplementary data on anchoring	SecSup	3
Other technical data	Year of construction	Year	8
	Natural wear (technical condition)	NatWea	5
	Repair factor	Repair	2
	Mining deformation resistance category	DRC	3
Mining data	Mining threat category of the terrain	MC	4
Damage	Damage category before impacts	DmgBef	4
	Damage category after impacts	DmgAft	4

**Fig. 2:**
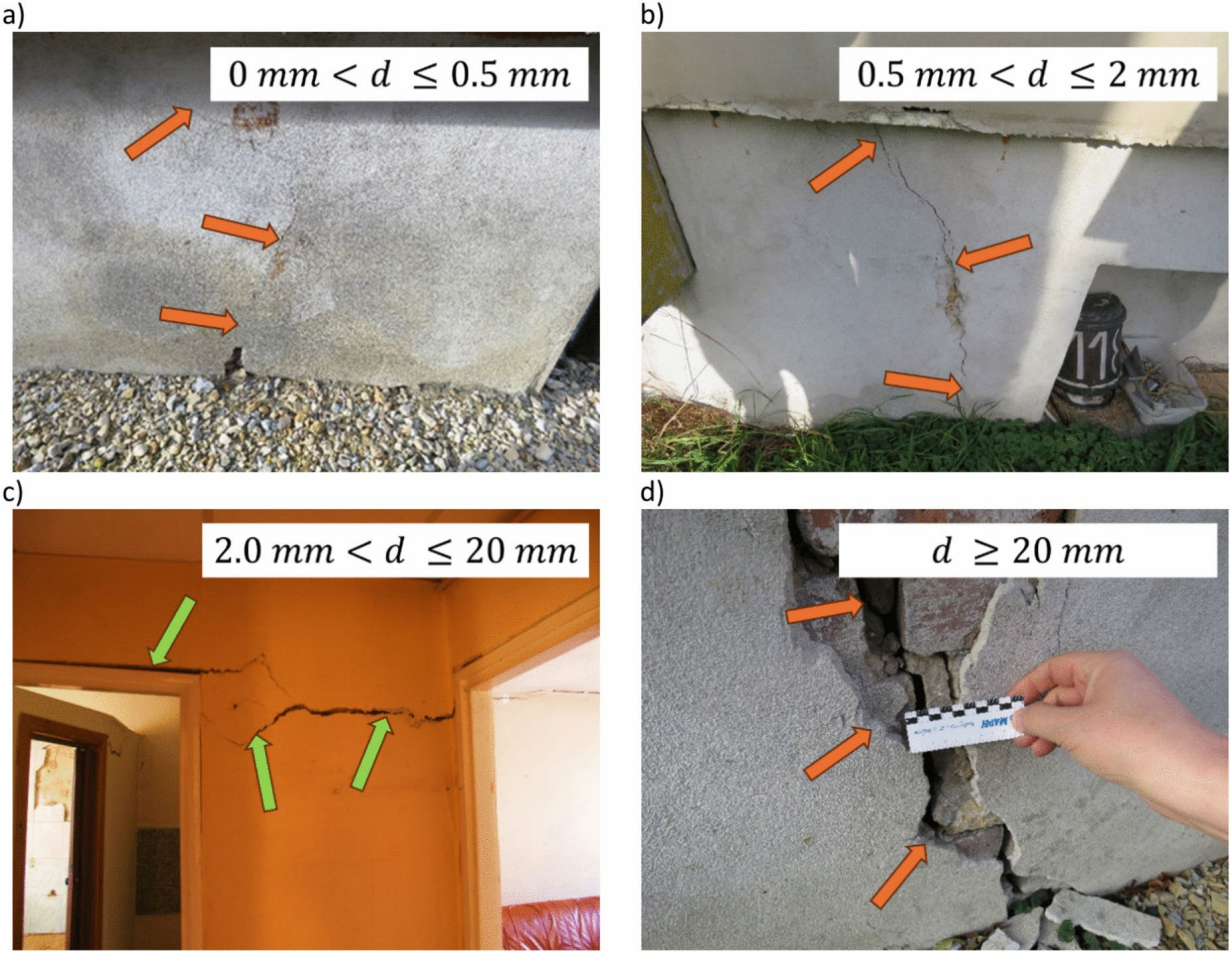
Examples of damage to a building classified as: (**a**) damage category 1; (**b**) damage category 2; (**c**) damage category 3; (**d**) damage category 4.

Damage detection was carried out during an inventory of the technical condition of a given building. A categorization of damage in accordance with the directives for assessing the hazard of buildings in mining areas was adopted. The scale described in^71^ is formulated in linguistic form and amounts, among other things, to determine whether the damage is to structural or secondary elements. There are 4 categories listed. However, it does not introduce a strict quantitative description concerning, for example, crack dilation. Therefore, in order to make the assessment of damage more unambiguous and meaningful, its range and intensity were made more detailed by introducing an additional parameter d (crack dilation). Thus, the assessment of the extent and intensity of damage consisted in determining what element of the building is damaged (structural or secondary element) and what value of dilation d the crack reaches. At the same time, the categorization of the level of damage was determined by the highest value of crack dilation found in the building. Figure 2 presents examples of damage and their qualification to the corresponding damage category. Meanwhile, the distribution of building damage categories from 2011 to 2017 is detailed in Table 2.

**Table 2:** Number of buildings with different damage categories from 2011 to 2017.

Damage category	Year of inventory				
	2011	2012	2014	2016	2017
1	18	19	18	20	8
2	49	115	89	108	79
3	22	42	63	55	50
4	6	8	16	12	12

**Fig. 3:**
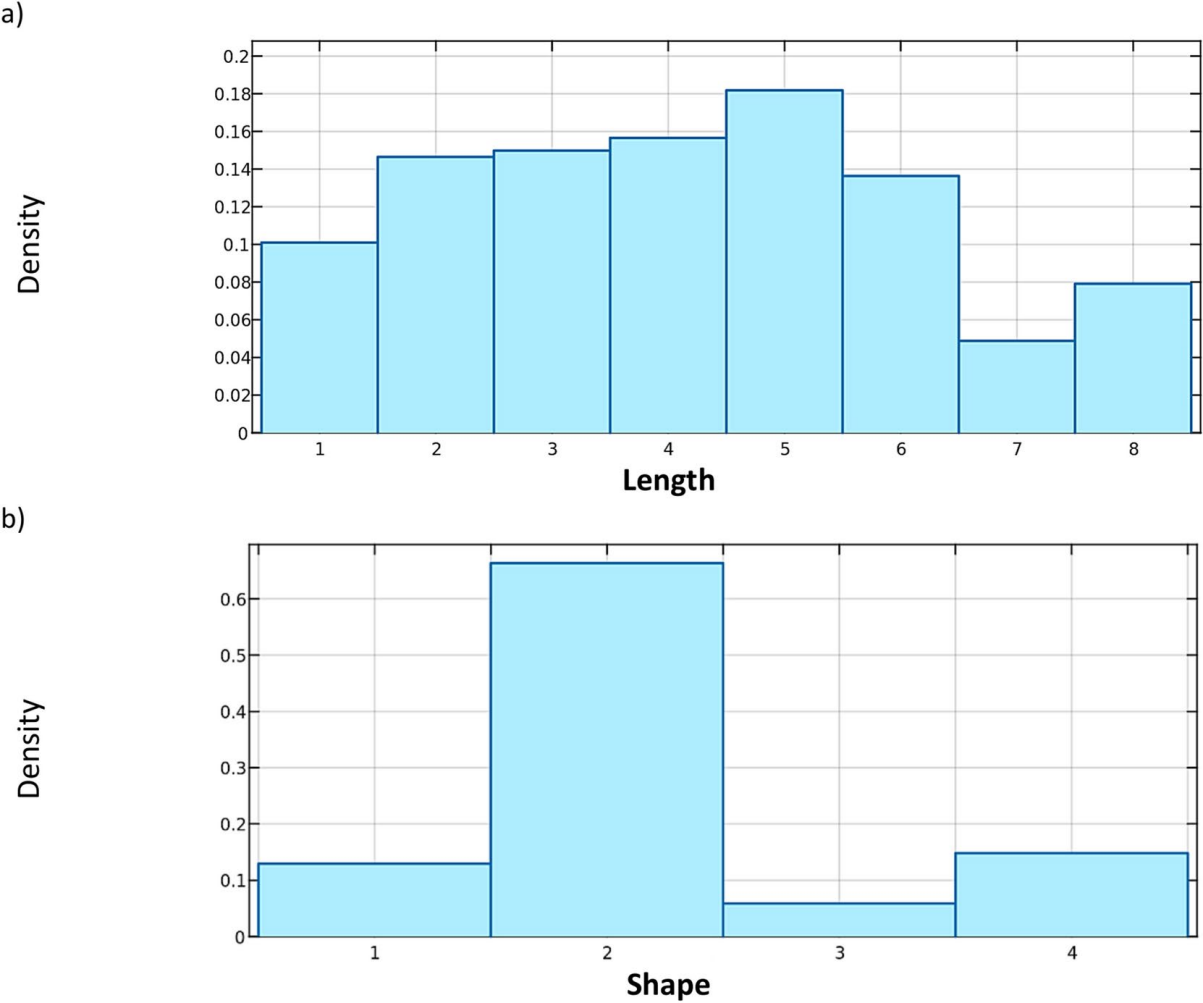
Examples of distributions of variables characterizing the geometry of buildings in the database: (**a**) **Length**, (**b**) **Shape**.

**Fig. 4:**
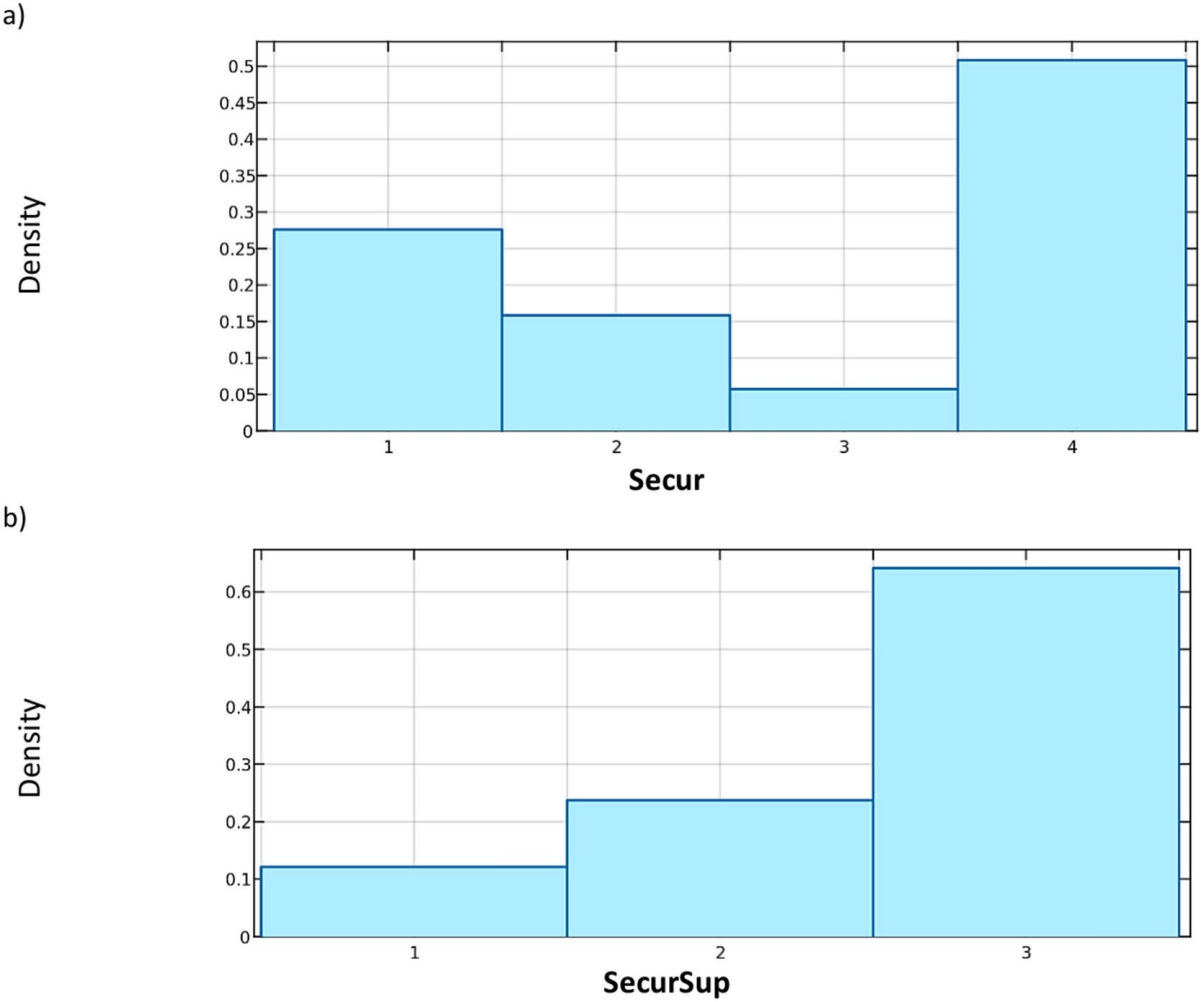
Quantitative distribution of variables describing the structural protection range: (**a**) **Secur**, (**b**) **SecSup**.

**Fig. 5:**
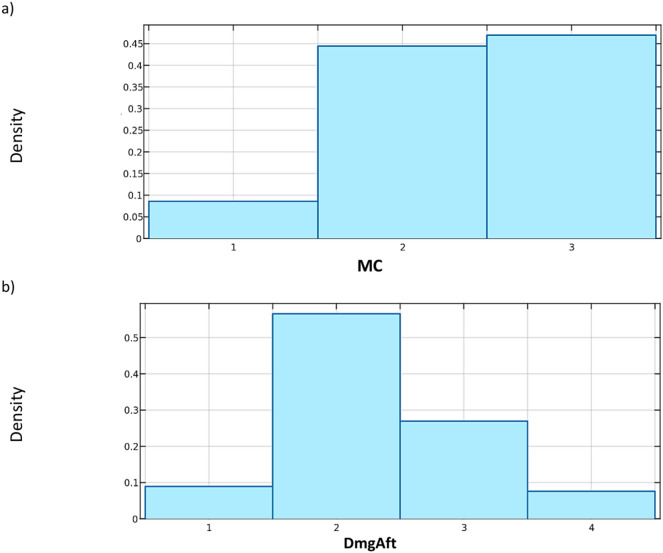
The distribution of variables describing the: (**a**) mining site hazard category **MC**, (**b**) damage range **DmgAft**.

The Table 1 describes each variable and the labels assigned to it. In addition, the distributions of the variables that characterize each group are presented in Figs. 3, 4 and 5.

The study used data from building inventories performed for the mining operations plan. As indicated earlier, each building was subjected to an assessment resulting from a survey of the exterior of the building, as well as its interior. This type of approach is aimed at detecting damage in the form of cracks and scratches, which do not cause a threat to the safety of the structure but contribute to a decrease in the utility standards of the given building. In addition, the problem is to identify properly the cause of the damage because they are often, in addition to mining impacts, the result of abandoned repair work, changes in usage, etc. Thus, this is a very time-consuming process and requires a lot of experience on the part of those performing it. Therefore, it can be assumed that this method of assessment is local and applies to a narrow area of land development, as well as being oriented toward detecting minor damage that does not threaten safety. Nevertheless, in the case of natural processes accompanied by much greater damage leading to a security threat that manifests itself to a far greater level, it is possible to clearly identify the causes of such an event. Then it is possible to successfully apply modern satellite imaging techniques, on the basis of which it is possible to construct probabilistic predictive models for assessing the threat of damage to buildings^72–75^.

### Characteristics of mining impacts

Mining impacts manifest themselves on the ground surface in the form of static and dynamic events. Both forms can threaten the safety of building structures. In most cases, however, they lead to damage that does not threaten safety but reduces the utility value of buildings. This, in turn, generates a serious socio-economic problem faced by residents and mining companies.

**Fig. 6:**
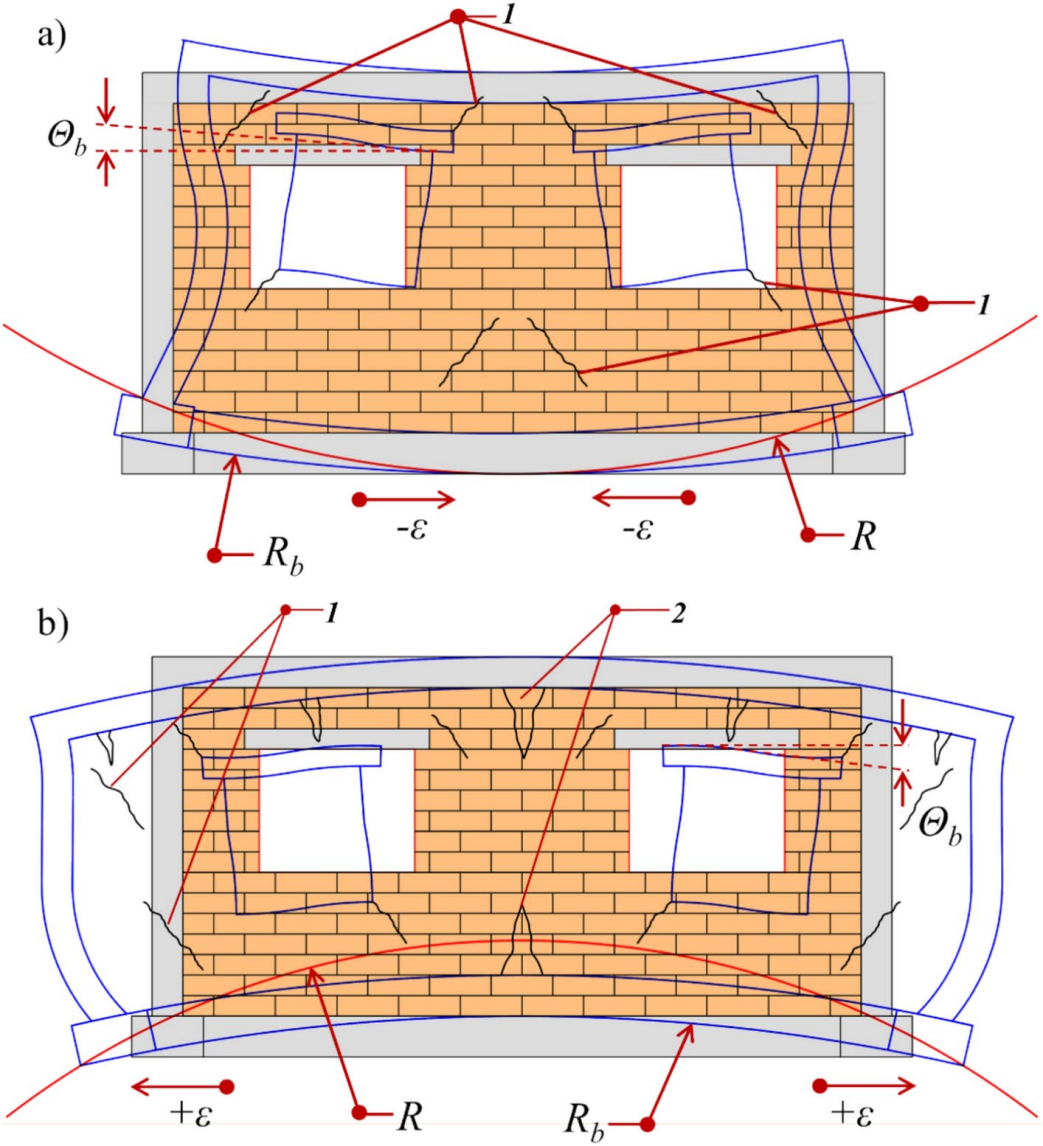
Diagram illustrating the morphology of damage based on the building’s location within the mining basin. *Θ*_*b*_*—*shear strains of building [mm/m], *R*_*b*_*—*radius of curvature (building) [km], *R—*radius of curvature ground) [km], + *ε—*horizontal tensile strains [mm/m], − *ε—*compressive horizontal strains [mm/m], *1—*diagonal cracks, *2—*vertical cracks^4^.

Static impacts include so-called continuous ground deformations. They represent a kinematic loading of the structure, leading to excessive force on its load-bearing elements and damage to secondary elements^6^. In engineering practice, the intensity of this type of influence is described by means of so-called mining ground hazard indicators. Here, vertical displacements (u), tilts (T), radius of curvature of the terrain (R) and horizontal strains (ε) are representative. These indicators are determined either by analytical calculations (in the case of designed mining operations) or by geodetic measurements (in the case of existing mining operations)^76–79^. The effect of a mining basin on a designed or existing building is illustrated in Fig. 6. As can be seen, a distinction must be made here between the nature of the prevailing impacts in terms of tension (convex basin) and compression (concave basin). This has important implications for the formation of building damage.

In the analysed area from which the collected building data comes, only static impacts of continuous deformation of the mining area were recorded. However, it should be noted that, in addition to such static impacts, there may be more violent processes resulting in so-called discontinuous deformations. With reference to the literature related to natural earthquakes, these are the same as the so-called PGD (Permanent Ground Deformations)^80–82^. They pose a greater threat to buildings due to the fact that they occur suddenly.

In addition to mining impacts, paraseismic phenomena, which are classified as dynamic impacts, should also be mentioned^8,10,83^. These are earthquakes of anthropogenic origin related to the process of underground fossil fuel extraction. They generate additional inertial forces in building structures, which can lead to a loss of load-bearing capacity of structural elements and contribute to damage. This study does not take mining tremors into account because they did not occur in the area where the underlying buildings are located.

From 2011 to 2017, the examined residential buildings were exposed to the impact of coal mining exploitation. The mining activities occurred at a depth ranging from 2.0 to 3.3 m, within the range of 625 to 805 m, specifically in the 503 and 510 decks.

To construct a database suitable for analysis, it was necessary to gather information on mining influences in the specific locations of individual buildings. To achieve this, data on the projected values of horizontal soil deformation (ε) were collected. The mining influences’ values were derived from regularly verified mining forecasts, approved based on results obtained from geodetic measurement lines along streets and scattered points on buildings. Utilising the impact of horizontal deformations, the resulting categories of the mining area were determined^84^ and illustrated in Table 3.

**Table 3:** The occurrence of mining area categories at the locations of the analysed buildings from 2011 to 2017.

Categories of mining area	Year of influences				
	2011	2012	2014	2016	2017
0	0	0	0	0	4
I	8	2	2	1	7
II	13	37	74	59	22
III	74	144	110	134	115

## Research methodology

Starting from scratch, any Bayesian network can be encoded as a tuple of the form^61,85^:

1 $$B= \left(G,{\varvec{\Theta}}\right)$$

where $G$—the Directed Acyclic Graph (DAG), ${\varvec{\Theta}}$—the set of parameters indicating the strength of the interactions between the variables encoded in the network structure.

The DAG structure consists of a set of nodes (X) and directed edges (E). The nodes represent the variables involved in describing the modelled process, while the edges are the links between the variables. Taking the above into account, the DAG structure can be succinctly described as:

2 $$G= \left({\varvec{X}},{\varvec{E}}\right)$$

where ${\varvec{X}}$—the set of variables constituting the nodes of a graph, ${\varvec{E}}$—the edges of the graph which are the links between the variables.

In probabilistic notation, the structure of a Bayesian network can be written as (47):

3 $$P\left(\mathbf{X}|G,{\varvec{\Theta}}\right)={\prod }_{i=1}^{N}P\left({X}_{i}|Pa\left({X}_{i}\right),{\Theta }_{{X}_{i}}\right)$$

where $G= G\left({\varvec{X}},E\right)$—the denotation used to describe the DAG structure, $\mathbf{X}= \left\{{X}_{1},\dots ,{X}_{N}\right\}$—the list of variables encoded in the nodes of the graph structure, $E$—the set of all edges, $Pa\left({X}_{i}\right)$—the set of parents of a vertex (variable) ${X}_{i}$, ${\Theta }_{{X}_{i}}$– the component values of the conditional probability table (CPT) between a vertex ${X}_{i}$ and its set of parents $Pa\left({X}_{i}\right)$, ${\varvec{\Theta}}=\left\{{\theta }_{{X}_{1}},\dots ,{\theta }_{{X}_{N}}\right\}$—the set of the component values of the conditional probability table (CPT) between given vertices and their set of parents.

The notation of the Bayesian network (BN) structure in the form (3) can be understood as the joint probability written in the domain of all variables ($\mathbf{X}= \left\{{X}_{1},\dots ,{X}_{N}\right\})$ under consideration^86^. Such a factorial notation of the joint probability is possible by applying the so-called chain rule and taking into account the conditional independence between the individual variables^87^.

Following the probabilistic notation further, it can be stated that the source of all the information held by the network is the so-called Conditional Probability Tables (CPTs). CPTs are located at each node and their size depends on the size of the node’s conditional parents set.

However, for the analysis of complex processes and phenomena, the creation of a Bayesian network in an expert manner via human knowledge is not possible. In order to meet tasks where the networks searched for are highly complex, a methodology for finding the optimal structure of Bayesian networks from data Bayesian Network Structure Learning (BNSL) was formulated.

At the core of the BNSL methodology is the formulation of a criterion indicating the extent to which a given network structure predicts the learning data set. In the field of methods for learning Bayesian networks from data, such criteria are most often so-called objective functions described, for example, in the form^86^:

4 $$sc\left( {G,D} \right) = \mathop \sum \limits_{i = 1}^{N} sc\left( {\left. {X_{i} } \right|Pa\left( {X_{i} } \right) = sm_{i} ,D} \right)$$

where $sm$—the adopted score metric to determine the level of explanation of the data by the different parts of the graph structure represented by the family variables.

As can be seen (4), the overall objective function for the entire Bayesian network structure is the resultant of the individual score metrics for the particular parts of the structure. On this basis, it is possible to estimate to what degree the individual network sections are able to explain the information contained in the learning data^64^.

In the issue of data-driven learning of Bayesian networks, strictly defined forms of metric scores are used. Among the most popular are, for example: Bayesian Information Criterion (BIC)^88^, Akaike Information Criterion (AIC)^89^, Log-Likelihood^85^ and Bayesian Dirichlet score metrics e.g.: BDeu^86^.

This formulation of the problem reduces Bayesian network learning to solving an optimisation problem via so-called scored-based methods^85,90,91^. Representative of this group of algorithms are, for example: Hill Climbing, Tabu^24^, K2^25^, A*^92^ or Simulated Annealing^93^.

As part of the present work, it was decided to use the Globally Optimal Bayesian Network learning using Integer Linear Programming (GOBNILP) approach^94^. This algorithm is also based on optimising the objective function and can be categorised as a score-based method.

At the core of the GOBNILP approach is a specific encoding of the Bayesian network structure. Key here are the so-called family variables with which the DAG structure can be encoded in zero–one notation (5)^64^.

5 $$f_{{X_{{i \leftarrow J}} }} = \left\{ {\begin{array}{*{20}l} 1 & {if\;{\text{J}}\;{\text{is}}\;{\text{the}}\;{\text{parent}}\;{\text{set}}\;{\text{for}}\;{\text{variable}}\;{\text{i}}} \\ 0 & {otherwise} \\ \end{array} } \right.$$

Adopting the principle of encoding the DAG structure in zero–one form using family variables imposes the need to consider consistency conditions. In general, two conditions are defined^64,95^. The first condition leads to the preservation of the internal consistency of the graph structure. Furthermore, it enforces that each variable, encoded as a vertex, has only one set of parents belonging to the set of all admissible combinations of the other variables (vertices). This condition, described according to relation (6), constitutes the first set of constraints, the so-called convexity constraints, which generates a set of constraints quantitatively equal to all vertices of the graph |V|.

**Fig. 7:**
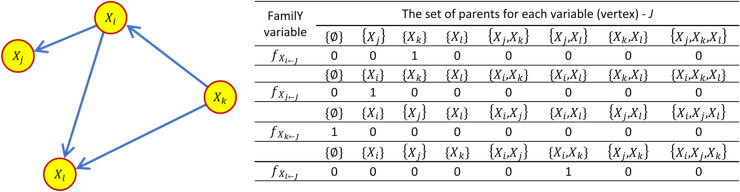
Diagram of the DAG structure and the corresponding coding system using so-called family variables according to the GOBNILP approach (50).

As can be seen in the diagram depicted in Fig. 7, a network structure correctly defined in this respect satisfies the condition set for each vertex.

6 $${\sum }_{J\in \mathcal{P}\left(i\right)}{f}_{{X}_{i\leftarrow J}}=1\hspace{1em}\forall i\in V$$

where $J$—the set of vertices representing parents for a vertex (variable) ${X}_{i}$, $\mathcal{P}\left(i\right)$—the set of all permitted combinations of vertices that may be parents for a variable (vertex) ${X}_{i}$

The application of the second condition (7) prevents a situation from arising in which cyclic links can appear in the graph. Under this condition, so-called clusters are considered. A cluster is understood as a set of at least two vertices that are separate from the graph. To satisfy this condition, it is necessary to indicate the number of vertices belonging to cluster C that do not have parents belonging to the same cluster. A graphical interpretation of this condition is presented in the work^4^.

7 $$\mathop \sum \limits_{i \in C} \mathop \sum \limits_{{J \in {\mathcal{P}}\left( i \right):J \cap C = \emptyset }} f_{{X_{i \leftarrow J} }} \ge 1\quad \forall C \subseteq V,\left| C \right| > 1$$

where $\text{C}$—a separated cluster consisting of at least two vertices of a graph.

Finally, according to the assumed convention of the creators of the GOBNILP algorithm, the original optimisation problem can be formulated, which can be written in the form (8)^95^. This formulation uses the aforementioned family variables and a user-defined metric score^85^.

8 $$\underset{G}{\mathit{max} \mathit{sc} }\left(G,D\right) =max{\sum }_{i\in V,J\in \mathcal{P}\left(i\right)}{{f}_{{X}_{i\leftarrow J}}sm}_{{X}_{i\leftarrow J}}\left(D\right)$$

9 $${f}_{{X}_{i\leftarrow J}}\in \left\{\text{0,1}\right\}$$

The formulation (8) together with the set of constraint conditions (6) and (7) supplemented by the assumption enforcing the integer value (integrality condition) of the solution (9) allow us to formulate the task that is the original optimisation problem in the GOBNILP algorithm^94^. The original optimisation problem formulated is a linear integer programming (ILP—Constraint Integer Programming) problem^62^. During the execution of the GOBNILP algorithm, a procedure called relaxation of the original IP problem to an LP linear programming problem is performed^63^. Using the constraints imposed on the clusters (7), which can be interpreted as so-called cutting planes for the LP problem, infeasible solutions are rejected. This procedure is carried out until, using the cluster constraints, no further possible cuts can be found that bring the problem closer to solution. In this case, a so-called branching procedure is initiated. Therefore, the GOBNILP algorithm implements the branching and cutting technique during its operation when solving linear integer programming problems^64^.

Originally, the GOBNILP algorithm was written in the C programming language. In this approach, an external computational solver, SCIP^96^, is used for solving integer programming problems with constraints (Constraint Integer Programming).

For the research, the results of which are described in this paper, it was decided to use a package in the Python programming language (pygobnilp^97^). In this instalment, the GOBNILP algorithm implements the use of the external computational solver Gurobi^98^.

## Results of the analyses and discussion

This research was aimed at obtaining the optimal structure of the direct acyclic graph (DAG), so that the resulting Bayesian network could make predictions of damage to buildings subjected to mining impacts. Variables were systematically grouped based on building characteristics and mining impacts. These variables were then categorised, and each fell under the multinomial classification.

All analyses were conducted utilising the pygobnilp package^97^ within the Python programming language. Before commencing the calculations, the data underwent preparation to facilitate the learning process following the guidelines tailored for Machine Learning methods and the specific recommendations for the GOBNILP algorithm^94^.

Initially, the dataset was partitioned into training and test subsets. During the separation process, the data was stratified based on variables describing damage intensity (**DmgBef** and **DmgAft**). This ensured that both training and test sets comprehensively covered the occurrence of all categories of damage intensity. The training set comprised 478 learning patterns, while the test set consisted of 116 patterns.

The second stage in preparing the training data involved establishing a baseline of expert knowledge regarding the relationships between the edges in the Bayesian network. The GOBNILP algorithm’s capability to impose constraints on individual edges discovered during the learning process was utilised for this purpose. This entailed determining disallowed connections in the Bayesian network. The knowledge was derived from the authors’ extensive experience gained through many years of on-site observations of buildings affected by mining operations. Only one forced connection was identified, leading from **MC** to **DmgAft**. Other connections leading from this variable were considered forbidden. The exceptions were variables describing the construction of the building, which may have resulted from the intensity of the prevailing mining conditions. The entire set of constraints imposed on the input dataset is pictured in Table 4. Care was taken not to introduce too many constraints, reducing to only the necessary ones. This was to leave more freedom during the discovery of the Bayesian network structure for the algorithm used.

**Table 4:**
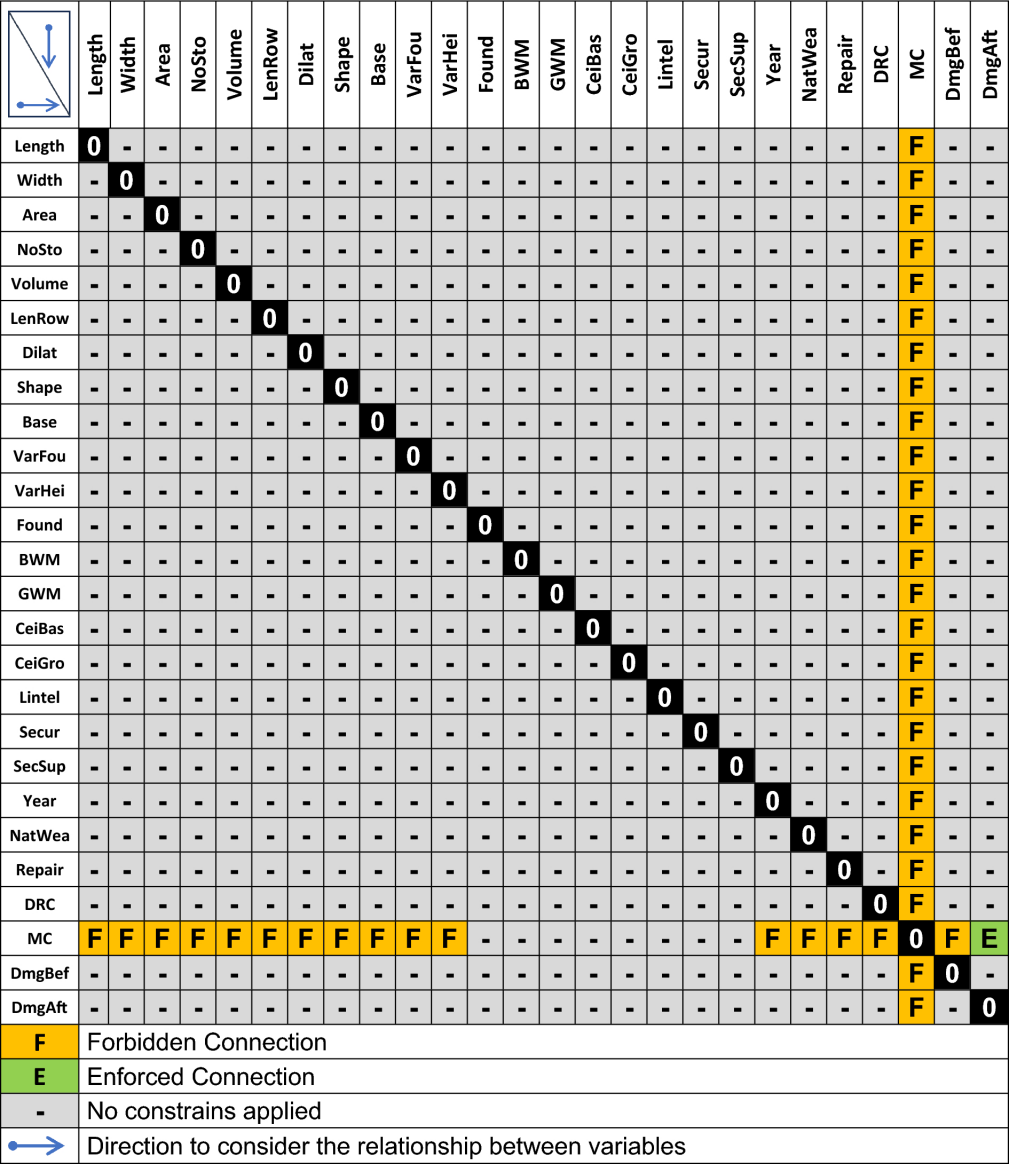
List of all applied forbiddances or enforcements imposed on connections in the network during learning.

In the preliminary stage, following a series of trial calculations, a constraint on the number of potential parents was introduced. This limitation aimed to address the generalisation properties of the models obtained during their testing on a set not used during training. Ultimately, the number of parents was restricted to six nodes (vertices). This limitation also had an impact on the computational time required for the calculations.

**Fig. 8:**
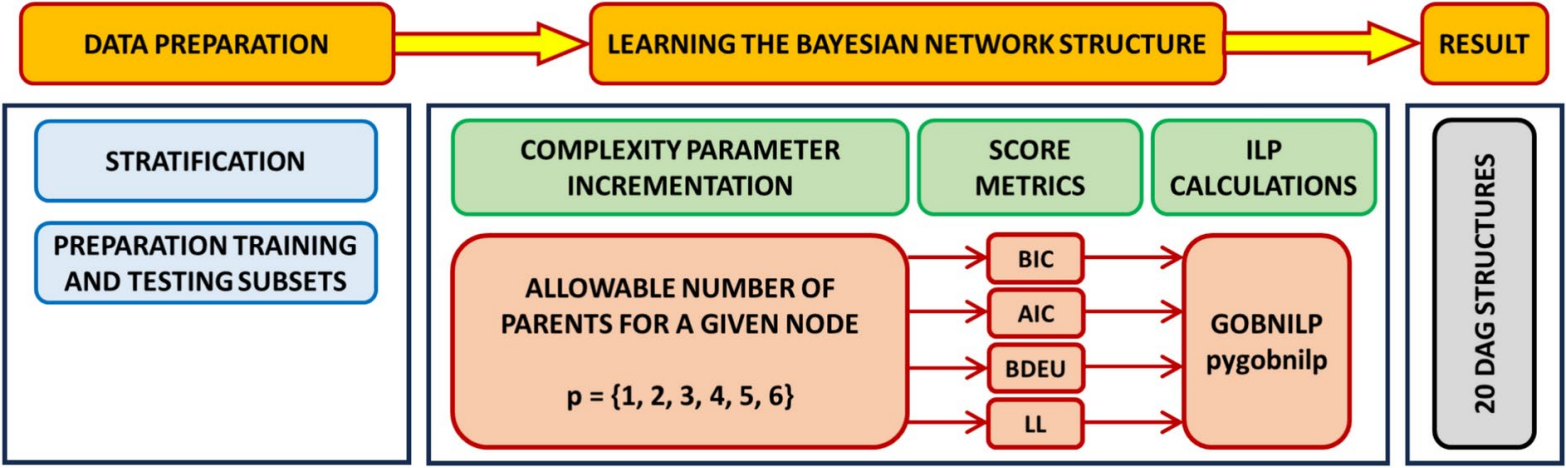
Schematic diagram of the numerical calculations undertaken.

Final calculations were made according to the GOBNILP algorithm. The prepared data set was taken into account, as well as assumptions imposed on certain links in the network structure. In order to take into account the influence of the metric scores on the calculations, the scope of analysis was extended. Four different scoring matrices were taken into account in the calculation range thus covered, namely: Bayesian Information Criterion (BIC)^88^, Akaike Information Criterion (AIC)^89^, Log-Likelihood^85^ and Bayesian Dirichlet equivalence score BDeU^64,86^. In addition, to ensure that the assumed network complexity was the optimal one, a complexity gradient was introduced. This consisted of introducing a different permissible number of parents for each node of the network. Thus, after multiple calculations, the optimal complexity of the resulting Bayesian network was determined for each metric score. The analysis procedure is presented in a simplified manner in Fig. 8.

**Fig. 9:**
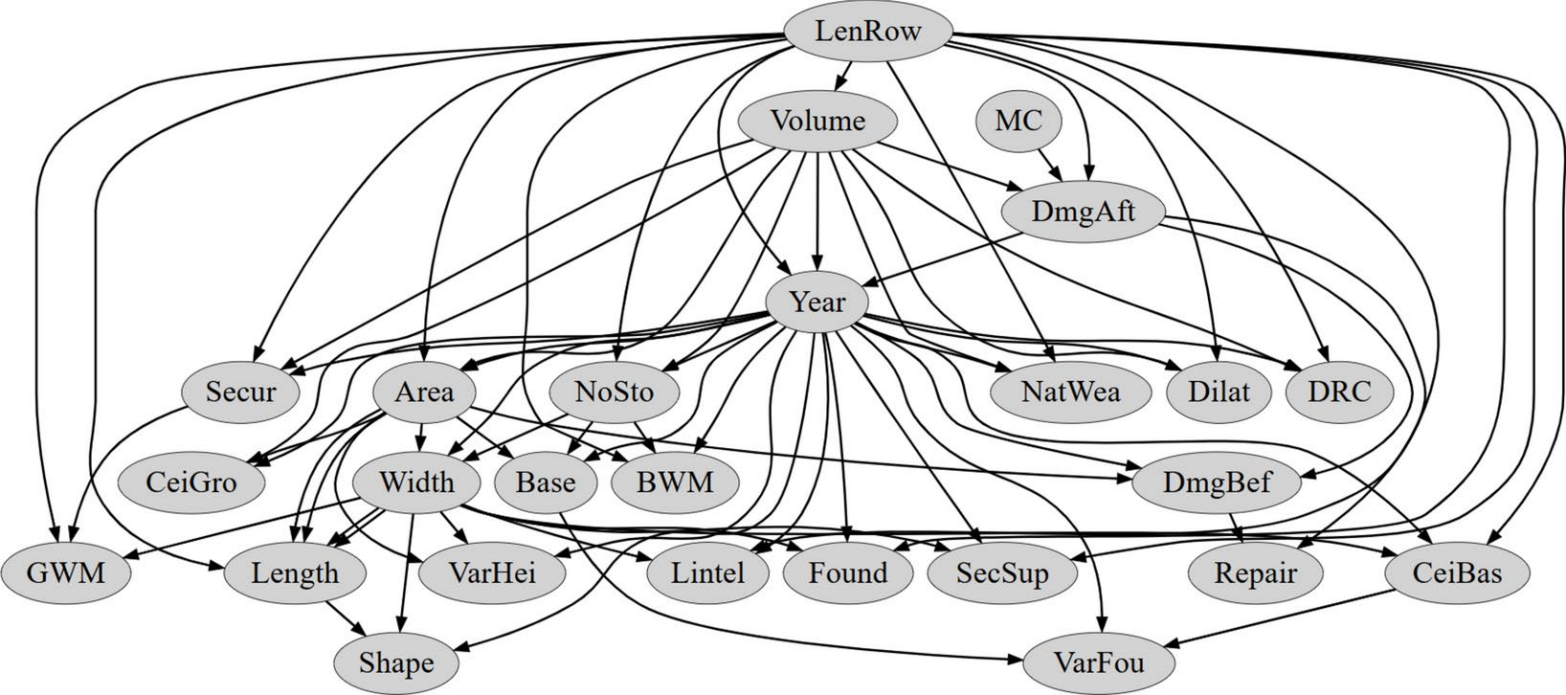
Diagram of the structure of the Bayesian network extracted using the LL scoring metric and complexity equal to ${\forall X}_{i}:\left|Pa\left({X}_{i}\right)\right|=4$.

**Fig. 10:**
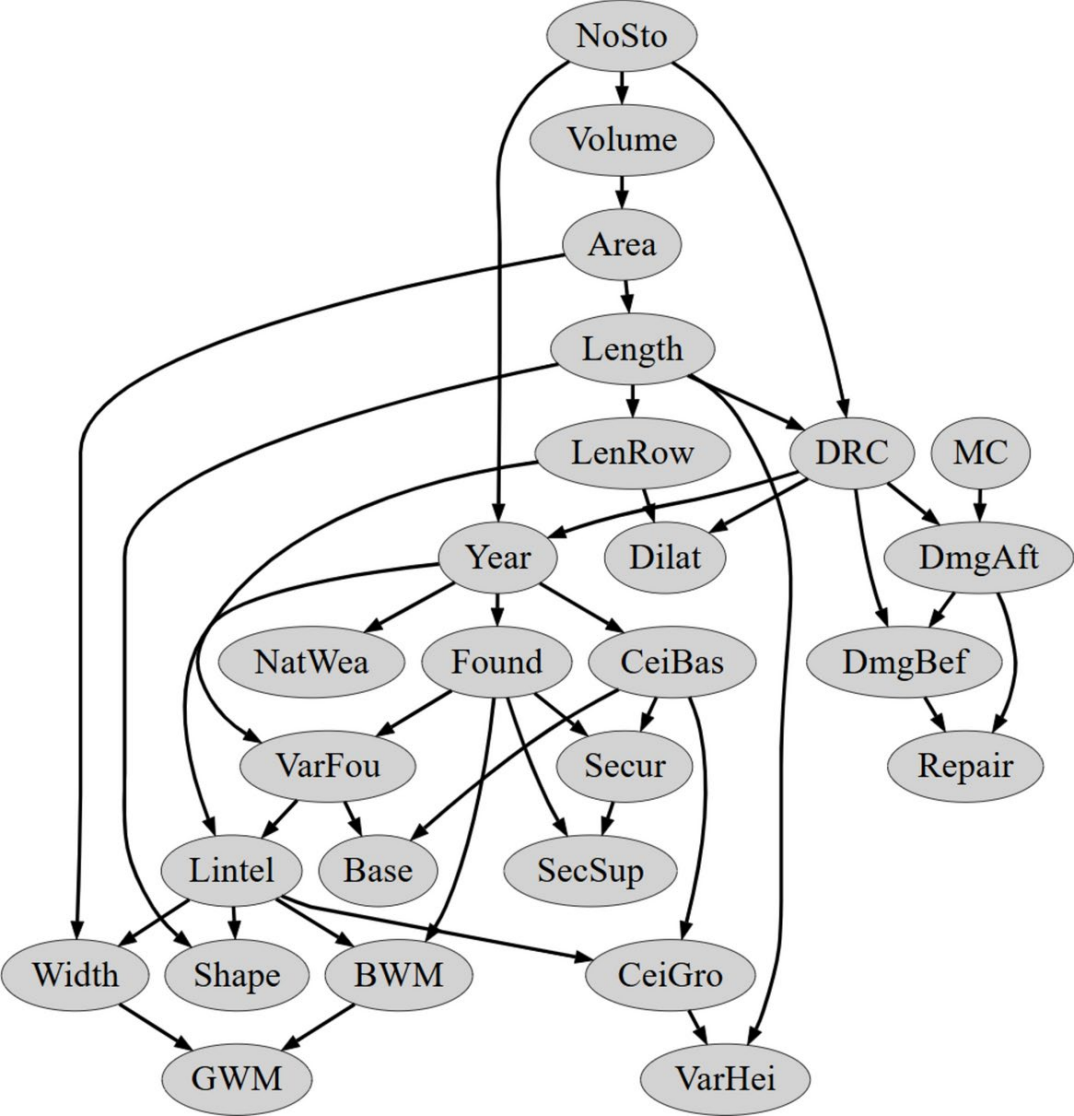
Diagram of the structure of the Bayesian network extracted using the AIC scoring metric and complexity equal to ${\forall X}_{i}:\left|Pa\left({X}_{i}\right)\right|=2$.

**Fig. 11:**
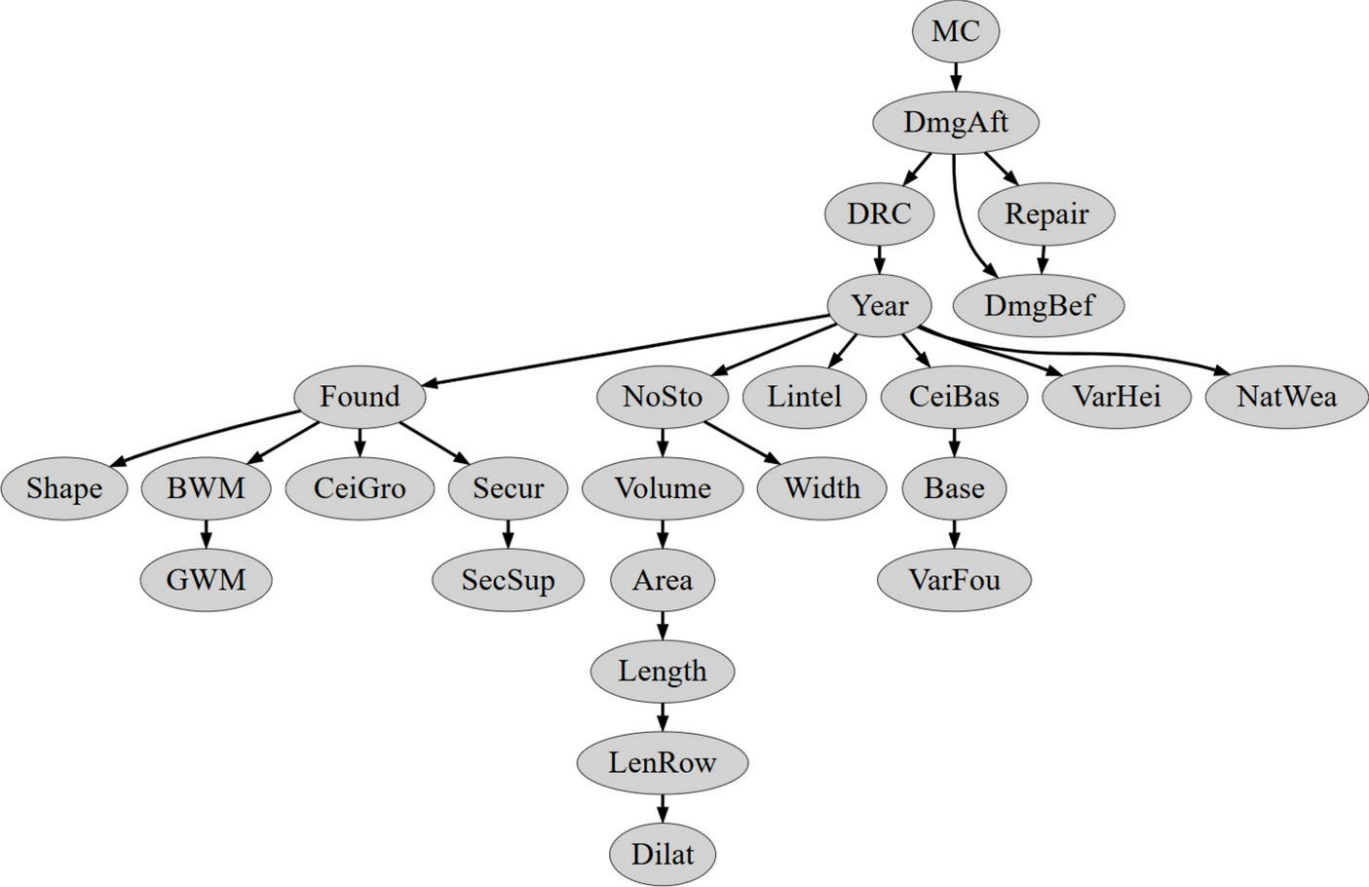
Diagram of the structure of the Bayesian network extracted using the BIC scoring metric and complexity equal to ${\forall X}_{i}:\left|Pa\left({X}_{i}\right)\right|=2$.

**Fig. 12:**
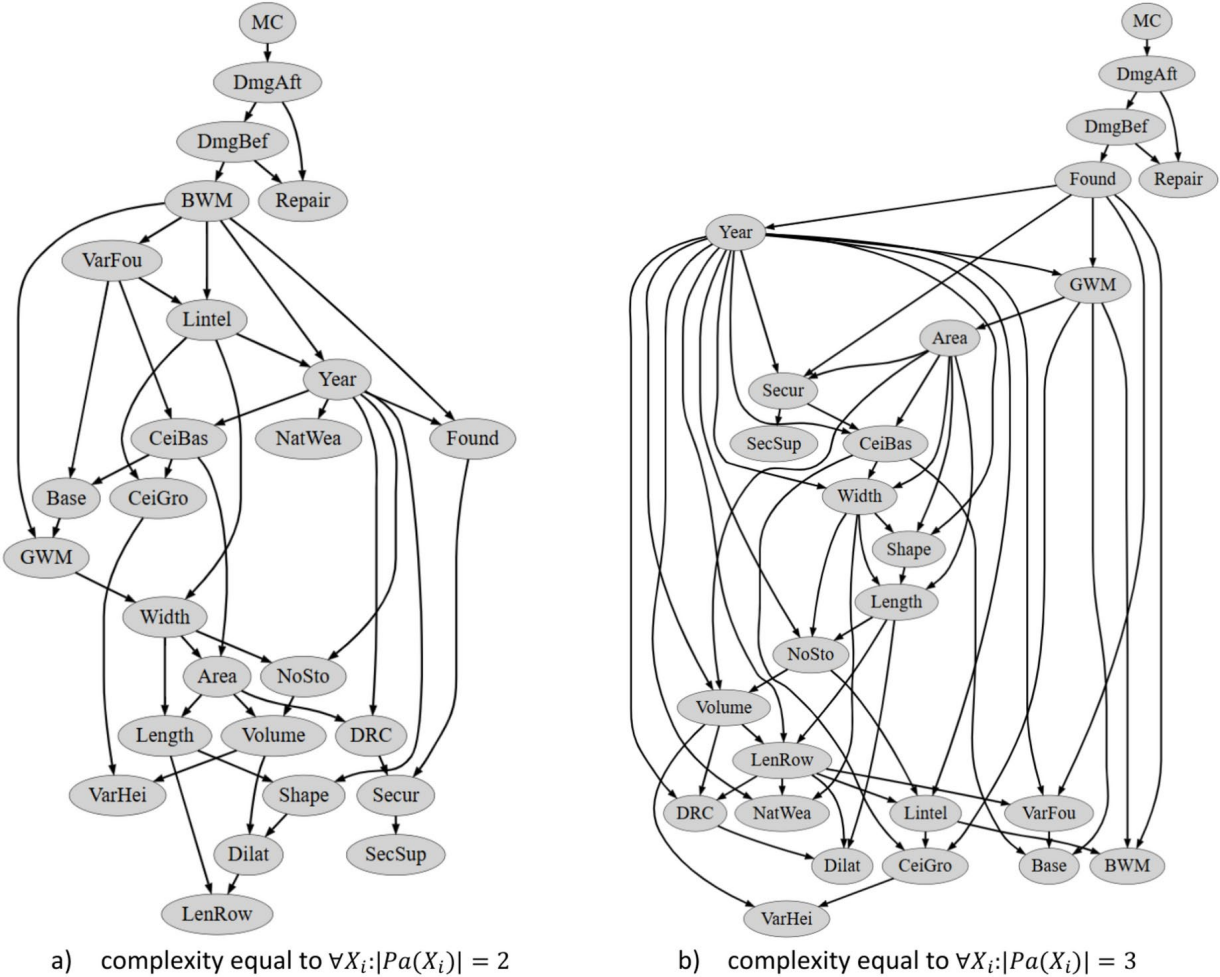
Diagram of the structure of the Bayesian network extracted using the BDeU scoring metric and complexity equal to ${\forall X}_{i}:\left|Pa\left({X}_{i}\right)\right|=2 and 3$.

As indicated in Fig. 8, by introducing different scoring metrics and increasing network complexity, a total of 20 different Bayesian networks were obtained. The results were verified on the basis of classification correctness for the learning and test set. Here, correspondence was verified only for the vertex with the coded variable describing damage intensity. A measure of the percentage of correctly classified patterns was used as a criterion for compliance. These results are presented in Tables 5, 6, 7 and 8 and Figs. 9, 10, 11 and 12. The results are grouped according to the scoring metric adopted. For each set, an attempt has also been made to indicate the change in quality of the network fit as a function of the complexity adopted at the start of the calculation. In addition, a relative measure indicating the generalisation properties of each trained network was introduced (R_e_). For this purpose, the absolute value of the difference of correctly classified cases for the training and test set was used. The results obtained were combined with those obtained for the training and test sets. The comparison measure was calibrated so that a high value of the measure would indicate over- or under-training of the Bayesian network being verified. Finally, in the selection of the final Bayesian network structure, those networks were selected for which the values of the adopted relative measure reached the lowest values.

**Table 5:** Distribution of classification accuracy for the emerged DAG structures (with respect to the variable **DmgAft**) depending on the complexity expressed by the permissible number of parents (AIC metric score).

Damage intensity	Number of parents set									
	2		3		4		5		6	
	Train set	Test set	Train set	Test set	Train set	Test set	Train set	Test set	Train set	Test set
**DmgAft**	77.41	75.86	77.41	77.86	77.41	77.86	77.62	77.86	77.62	77.86
R_e_	1.55		1.55		1.55		0.45		0.45

**Table 6:** Distribution of classification accuracy for the emerged DAG structures (with respect to the variable **DmgAft**) depending on the complexity expressed by the permissible number of parents (BIC metric score).

Damage intensity	Number of parents set									
	2		3		4		5		6	
	Train set	Test set	Train set	Test set	Train set	Test set	Train set	Test set	Train set	Test set
**DmgAft**	76.57	77.59	76.57	76.72	76.57	77.59	76.57	77.59	76.57	77.59
R_e_	1.02		0.15		1.02		1.02		1.02

**Table 7:** Distribution of classification accuracy for the emerged DAG structures (with respect to the variable **DmgAft**) depending on the complexity expressed by the permissible number of parents (LL metric score).

Damage intensity	Number of parents set									
	2		3		4		5		6	
	Train set	Test set	Train set	Test set	Train set	Test set	Train set	Test set	Train set	Test set
**DmgAft**	79.92	79.31	91.89	91.40	91.21	92.24	91.21	92.24	91.21	92.24
R_e_	0.61		0.49		1.03		1.03		1.03

**Table 8:** Distribution of classification accuracy for the emerged DAG structures (with respect to the variable **DmgAft**) depending on the complexity expressed by the permissible number of parents (BDeU metric score).

Damage intensity	Number of parents set									
	2		3		4		5		6	
	Train set	Test set	Train set	Test set	Train set	Test set	Train set	Test set	Train set	Test set
**DmgAft**	77.41	76.72	77.41	79.31	76.98	76.72	76.35	77.58	76.35	77.58
R_e_	0.69		1.90		0.26		1.23		1.23

Of all the Bayesian network structures obtained, the best were those using the LL scoring metric. In turn, the best of the family of networks created for the LL scoring metric turned out to be the one for which the number of parents equal to ${\forall X}_{i}:\left|Pa\left({X}_{i}\right)\right|=4$ and more was assumed. However, bearing in mind the desire to obtain the simplest network, the final structure chosen was the network with the LL scoring metric for the maximum number of parents in each node equal to 3. The results for this network are summarised in Table 7, while a diagram of the structure is presented in Fig. 9.

Other network structures that emerged achieved good properties in terms of the quality of the prediction fit to the benchmark data, achieving in all cases around 76% agreement with the benchmark data. The results for networks obtained using the AIC, BIC and BDeU scoring metrics are summarised in Tables 5, 6 and 8. Thus, the best network obtained using the AIC measure was a structure with a complexity of ${\forall X}_{i}:\left|Pa\left({X}_{i}\right)\right|=2$. Here, by increasing the number of parents allowed, it was not possible to obtain a network of higher complexity. The situation is similar for networks obtained using the BIC scoring metric. Here, the best network turned out to be the one for which the number of parents for each node was, as in the case of the AIC scoring metric, ${\forall X}_{i}:\left|Pa\left({X}_{i}\right)\right|=2$. Diagrams of the emergent network structures for the adopted AIC and BIC scoring metrics are summarised in Figs. 10 and 11.

The last BDeU scoring metric used allowed the optimal network structure to emerge with a complexity ${\forall X}_{i}:\left|Pa\left({X}_{i}\right)\right|=3$. Nevertheless, there is only a subtle improvement in the prediction error here for the test set relative to the network with a complexity of ${\forall X}_{i}:\left|Pa\left({X}_{i}\right)\right|=2$ (cf. Table 7). A view of the structure of these two networks for complexity equal to $\forall X_{i} :\left| {Pa\left( {X_{i} } \right)} \right| = 2\; {\text{and}}\; 3$ is presented in Fig. 12.

Figure 9 shows that the variables **MC**, **Volume**, and **LenRow** have a significant impact on the state of damage. Reasonable connections of the variables **Volume**-**Area**-**Width**-**Length** to each other are also visible, but the connections of some variables like **Year**-**Shape** or **Width**-**Found** are questionable.

Figure 10 illustrates that variables such as **MC**, **DRC**, **Length**, **Area**, **Volume**, and **NoSto** exert a substantial influence on the damage state. Clear connections between construction and geometrical variables are evident. However, certain connections, such as **Lintel**-**VarFou** or **Length**-**VarHei**, raise questions about their validity.

Figure 11 indicates that the only variable significantly affecting the damage state is **MC**, which does not align well with experiences in mining areas. While there are clear connections between construction and geometrical variables, certain links, such as **Found**-**Shape** or **Year**-**NoSto**, cast doubt on their validity.

Figure 12 indicates that again the only variable significantly affecting the damage state is **MC**, which does not align well with experiences in mining areas. There are some understandable connections between construction like **Found**-**Secur** or **CeiBas**-**CeiGro** and geometrical variables like **Width**-**Length**-**Area**-**Volume** connections. Unfortunately, certain links, such as **Lintel**-**Width** or **Year**-**NoSto**, are hard to understand.

To sum up, the obtained classification accuracy of over 76% allows for the effective use of this methodology to conduct research on the occurrence of damage to buildings exposed to the influence of mining. The best results of over 91% were obtained for LL metric score and at least 3 parents, which indicates the model’s above-average predictive abilities.

A very important advantage of Bayesian networks is the presentation of network results in the form of directed graphs. The preliminary analysis of the obtained graphs (Figs. 9, 10, 11 and 12) showed that the GOBNILP network training method proposed many justified and naturally supported connections of input variables. The analysis also showed that some of the obtained networks greatly narrow the contribution of input variables to the damage status of the building **DmgAft**, sometimes to only one variable.

## Conclusions

This paper addresses the problem of assessing damage to masonry buildings subjected to repeated impacts induced by underground mining activities. Due to the large number of factors describing the damage process, it was decided to use Bayesian network methodology. For this purpose, with a dataset at our disposal, the GOBNILP tool was used to detect the Bayesian network structure from the data. The analyses were carried out successively in order to computationally capture the impact of network complexity on the quality of the model fit to the reference data. By performing complexity gradations and using different score metrics, Bayesian network structures were obtained and discussed in terms of prediction quality and causal links. In the case of cause-effect relationships, the focus was on the authors’ own experience and long-term observations of the damage process of masonry buildings subjected to the influence of mining exploitation. According to the study, the best network that emerged was the one for which the score metric Log-Likelihood was applied. This network obtained the best prediction properties for both the training set and the test set. The other network structures presented also had good predictive properties for the information contained in the reference data. As the Bayesian network structures emerging from the learning process are different to each other, a sensitivity analysis of the connections between network nodes is planned for further research. This will require further data collecting and additional calculations.

At the present time, the emerged structure of the Bayesian network fulfils the assumptions made at the beginning of the research regarding the prediction of damage intensity in the case of the occurrence of mining impacts and the diagnosis of the damage state that has occurred. Assuming that such a network plays the role of a decision support system, it is possible to apply and implement it for use in the case of the necessity to assess the damage to a large number of buildings affected by mining exploitation. In this way, it can serve as an effective tool in the interdisciplinary area of hazard assessment at the borderline of civil engineering, environment, and mining.

An disadvantage associated with the used BNSL methodology is the obtaining of different structures depending on the adopted score metric function. In addition, the process of structure extraction for a large number of variables implies a large computational difficulty. To protect against an increase in computational complexity, the complexity of the network expressed in the number of acceptable parents for a given node should be incremented. The advantage is the ability to take into account the expert’s knowledge of the connections in the network that should exist and those that are not allowed to occur. This makes the process of extracting structure from data can be called hybrid.

While assembling this article, other newly developed approaches to learning the structure of Bayesian networks from data were noticed. Very interesting is the Differentiable Bayesian Structure Learning approach described in the paper^99^. Another very attractive approach is the use of Generative Flow Networks in the learning process of Bayesian networks^100^. Another iteration of the methodology for learning Bayesian networks from data is offered via the modern RBNets technique of using deep learning with reinforcement^101^. This type of solution will be the subject of further analysis undertaken by the authors’ team in the future.

The research was directed toward the creating of a damage prediction model for buildings of masonry construction located in a strictly defined mining area. Therefore, it must be emphasized that such a model will have limited applicability, which can only be applied to masonry buildings. Wishing to consider another type of building structures, it is necessary to first create a dedicated database from scratch, which is the basis for the procedure of extracting the structure of the Bayesian network from the data. This type of research is being carried out in parallel by the authors’ team and concerns multi-story residential buildings made of reinforced concrete large panel technology^3,102^. In addition, it should be emphasized that the locality of the model also refers to the location of the mining area from which the learning data is derived. Undoubtedly, the fact that there are different intensities of continuous deformation in different areas and, in addition, the possibility of mining tremors in these areas as well, may determine the different course of the damage process in buildings. This statement can be related to the phenomenon of the difference in the dynamic resistance of building structures located in areas with a different intensity of mining tremors^103,104^.

## Data Availability

The data presented in this study are available on request from the corresponding author. The data are not publicly available because they were taken from studies carried out for private enterprises.
